# Compassionate pedagogy for neurodiversity in higher education: A conceptual analysis

**DOI:** 10.3389/fpsyg.2023.1093290

**Published:** 2023-02-16

**Authors:** Lorna G. Hamilton, Stephanie Petty

**Affiliations:** School of Education, Language and Psychology, York St John University, York, United Kingdom

**Keywords:** neurodiversity, higher education, compassion, double empathy, Universal Design for Learning

## Abstract

The neurodiversity paradigm challenges pathologising accounts of neurodevelopmental differences, including autism, attention deficit disorder (ADHD), dyslexia, developmental language disorder (DLD) and others. From a neurodiversity perspective, these differences in the way people perceive, learn about and interact with the world are conceptualised as naturally occurring cognitive variation, akin to biodiversity in the natural environment, which may bring unique strengths and challenges for individuals. An implication of this approach is that interventions designed to create contexts in which neurodivergent people can thrive are needed, in addition to those that seek to ameliorate individual-level difficulties. In this conceptual review, we consider how higher education can offer a context in which cognitive diversity can be noticed, welcomed and accepted with warmth. In universities, neurodiversity is one dimension of difference within an increasingly diverse student population, which overlaps – but is not synonymous – with disability. We argue that improving experience and outcomes for neurodivergent students should be a priority for universities aiming to produce graduates equipped to tackle the complex problems of contemporary society. Drawing on the foundational principles of compassion-focused psychological therapies, we consider how compassion can be enacted within interpersonal interaction, curriculum design, and leadership culture in universities. We apply the insights of double empathy theory to the problem of overcoming barriers of difference in the classroom. Finally, we make recommendations for Universal Design for Learning (UDL) and strengths-based pedagogical approaches, which create a fit-for-purpose educational environment for the widest possible range of learners. This realignment with the neurodiversity paradigm offers an antidote to bolt-on provisions for students who differ from the neuro-normative, and might enable neurodivergent thinkers to flourish within and beyond higher education.

## 1. Introduction

In recent years, widening access to higher education in the United Kingdom and globally has created a larger, more diverse student population (Calderon, 2018; Higher Education Statistics Agency [HESA], 2022). Neurodiversity is a dimension of difference that has received relatively little attention in the higher education pedagogical literature, despite there being increasing numbers of neurodivergent students attending university internationally (Pino and Mortari, 2014; Bakker et al., 2019). Neurodiversity can be understood as naturally occurring variation in the ways that humans perceive, experience and interact with the world, encompassing neurodevelopmental differences such as autism, attention deficit disorder (ADHD), dyslexia, developmental language disorder (DLD), dyscalculia, and developmental co-ordination disorder (DCD) (see Dwyer, 2022 for a discussion of more or less inclusive definitions of neurodiversity). Robust data on outcomes for neurodivergent students in higher education are not currently available; however, existing studies indicate that wellbeing and employment outcomes tend to be poorer in this population in comparison with their peers (Anderson et al., 2017; Allen and Coney, 2019; Bayeh, 2022).

In this conceptual analysis, we draw on psychological understandings and applications of compassion to consider how universities can support neurodivergent thinkers to thrive. Cognitive diversity is essential in the search for solutions to the complex problems facing the world (Sulik et al., 2021), and therefore universities must prioritise improved outcomes for neurodivergent students. We propose that this can be achieved by adopting compassionate pedagogies, including universal design for learning and strengths-based approaches (Gibbs, 2017; Elsherif et al., 2022). Neurodivergent students, like all students, deserve fit-for-purpose learning experiences, and should not hold responsibility for educating their educators about their differences. The structures and policy context of higher education can act as a disincentive to personalised learning and teaching (Waddington, 2017). Nonetheless, compassion-informed pedagogy, in which educators actively recognise the particular struggles that students face and seek to mitigate them, can be transformative for neurodivergent learners.

## 2. Neurodivergent students at university

Obtaining a clear picture of outcomes for neurodivergent students at university is complex, not least because many do not disclose their diagnosis, and others may not have a formal diagnosis when they commence their studies (McLeod et al., 2019; Clouder et al., 2020). Evidence to date, which often relates specifically to autism, dyslexia and/or ADHD, suggests that academic attainment can be good, if students are well supported to complete their studies (Richardson, 2009; Fabri and Andrews, 2016; Richardson, 2017). However, attrition rates are relatively high in this population, with poor mental health being just one of many contributing reasons (Van Hees et al., 2015; Ghisi et al., 2016; Griffiths et al., 2019).

It is not difficult to imagine some of the contextual factors that underpin poor retention of neurodivergent students, some of which are discussed here. First, the ‘hidden curriculum’ at university – that is, the unwritten, and sometimes unintentional, institutional expectations of how students will behave, study, and interact, which are not explicitly taught – tends to exclude minoritised groups from reaching their potential (Sulaimani and Gut, 2019). Second, neurodivergent students often come to university with a negative schema of education following their experiences at school (Lithari, 2019; Mesa and Hamilton, 2022a,b). Experiences of non-accepting environments in earlier education can have long-lasting impacts, and can contribute to a negative sense of self, affected wellbeing and reduced quality of life for older students and adults (Hong et al., 2016). Third, considering some of the specific challenges for neurodivergent students, the near total reliance on written forms of assessment in many degree programmes is a disproportionate barrier for dyslexic students (Jacobs et al., 2022). ADHDers may find managing independent self-study and processing critical feedback particularly difficult (for an overview of rejection sensitivity in ADHD, see Bedrossian, 2021). For autistic students, achieving a sense of belonging in the university community may be a key challenge: social situations can be overwhelming but unavoidable, and socialising in different ways with peers can attract bullying or result in feelings of loneliness (Bauminger et al., 2003; Gurbuz et al., 2019). Students often mask their differences, by suppressing natural neurodivergent responses and/or learning and imitating ‘neurotypical behaviours’ in an attempt to avoid negative responses from others. Masking difference can lead to exhaustion and burnout, disconnection from one’s identity, and psychological distress (Pearson and Rose, 2021).

We suggest that current educational contexts largely illustrate a conditional view of an acceptable student, i.e., a student is acceptable only when fitting to a neurotypical standard (Spaeth and Pearson, 2021). Difference is often construed as negative, and only rarely understood as demonstrating novelty, originality or excellence in academia. For many neurodivergent students, experiences of personal confusion, navigation of multiple identities and labels through which to view themselves, and experiences of bullying and marginalisation, are all threats to holding a robust, compassionate view of the self within a university environment. Understandably, students often assume high personal responsibility for trying to make a success of their education. Many neurodivergent students describe having to act as self-advocates in order make others understand their difference, which can contribute to disenfranchisement from university communities (Fabri and Andrews, 2016; Elias and White, 2018).

## 3. Contextual approaches to neurodiversity

Research into autism and other neurodevelopmental differences is undergoing a paradigm shift, away from medical models and towards neurodiversity approaches (Dwyer, 2022; Pellicano and den Houting, 2022; for alternative perspectives and critiques of the neurodiversity paradigm, see Nelson, 2020; Singer et al., 2022). One key limitation of the medical paradigm in this field is its narrow focus on the individual as the unit of study, with the aim of identifying and ameliorating deficits. In contrast, research from a neurodiversity perspective foregrounds the person-in-context; *impairment* at the individual level is acknowledged where it exists, but *harm* associated with impairment is seen as a function of the interaction between the person and their environment (Singer, 2017; Chapman, 2021). For instance, hyperactivity may be harmful for a child in a school with strict rules on sitting still in class, but harmless for that same child participating in an outdoor education class. It follows that interventions targeting the contexts in which neurodivergent people live, study and work are needed, in addition to existing interventions aimed at improving skills (or, more controversially, modifying behaviours) in individuals. Higher education is one such context, where structures, processes and pedagogies can be designed to be more inclusive for neurodivergent students and staff.

The social environment is central to any educational setting: learning depends on the interactions that take place between the student, their peers, academic and professional staff. Here the double empathy problem (Milton, 2012) is pertinent. The double empathy problem refers to the reciprocal deficits in understanding that can occur between people who hold different norms and expectations of each other. Where communication preferences and sensory sensitivities vary across neurotypes, this problem can be particularly pervasive. A growing body of experimental research supports the premises of the double empathy problem; for example, neurotypical people are quick to form negative impressions of autistic people on the basis of scant information (Sasson et al., 2017). Furthermore, certain types of communication, including information sharing, can be more successful within same-neurotype than cross-neurotype pairs (Crompton et al., 2020). When people see the world from different perspectives, insight and compassion into each other’s difference is more helpful than assuming that one experience is normative, while the other deviates from the norm.

In order to overcome double empathy barriers in the classroom, as educators we must reflect critically on our assumptions and practice. This could mean consciously avoiding the interpretation of students’ behaviour from a default neuro-normative perspective. What could be the underlying reasons for a student keeping their eyes closed in class, or taking their seat at the last moment rather than queueing or conversing with peers? It could involve examining where our own knowledge of neurodiversity comes from and critically assessing the potentially stigmatising assumptions that we hold. Carefully considering the language that we use when discussing difference can be powerful in creating a more neurodiversity-inclusive learning environment. Avoiding the language of pathology (e.g., symptoms, comorbidities, high/low functioning) in favour of more neutral alternatives (e.g., characteristics, co-occurring considerations, individual abilities or support needs) is a simple modification that can have a meaningful impact on students’ sense of place in the classroom, and in turn, how the education community of peers and staff make sense of difference (Bottema-Beutel et al., 2021). There is good evidence, at least in the Anglophone world, that many autistic people prefer identity-first language (e.g., autistic student) over person-first language (e.g., student with autism), which can imply that the autism is separable from the person (Kenny et al., 2015; Taboas et al., 2022; see Buijsman et al., 2022 for different terminology preferences in a Dutch sample). Analogous identity-first terminology for other neurodevelopmental differences is not yet clear or settled, but the language of neurodiversity is constantly evolving. Initiating open dialogue with neurodivergent students about their language preferences can help to mitigate the double empathy problem in the classroom.

Unfortunately, in order to access educational support at university *via* disability services, students often have to use the terminology of the medical model, emphasising their diagnosis and deficit and downplaying their strengths. Negotiating this dual reality is a current tension for many neurodivergent students, which signals the need for change towards universal design approaches. Implementing the insights of the neurodiversity paradigm in universities means going beyond simple adaptations or add-ons to current practice (Petty et al., 2023). Currently the onus is too often on neurominority students to find a longer way around to meet neuro-normative expectations. To transform practice, learning and teaching must be designed for a neurodiverse student body, and learning contexts created in which neurodivergent students are seen, understood and enabled to thrive. Here, educators can harness the potential of compassionate pedagogy.

## 4. Learning from applications of compassion in other fields

Various definitions of compassion exist in the psychological and philosophical literatures. Most share two key components: (1) the propensity to notice suffering in oneself and others (*all* others, regardless of minority status) without negative judgement and (2) the motivation to act to prevent or alleviate suffering (Halifax, 2012; Gilbert, 2019). Nussbaum (2001) emphasises both its cognitive, evaluative nature and its teachability; we learn compassion through experience with diverse others (in real-world interactions and in simulated, fictional social worlds) and subsequent reflection on those experiences. In Nussbaum’s conceptualisation, a prerequisite for compassionate responses to another’s humanity is the acknowledgement of one’s own human vulnerability.

Concepts of compassionate others, compassionate memories and compassionate spaces underpin many psychological therapeutic practices, notably within compassion-focused therapy (Gilbert, 2007). For example, a client experiencing high anxiety and shame might be encouraged to explore early memories of these emotions, to notice where judgements and expectations from others caused a sense of threat, while working therapeutically to harness the soothing potential of compassion. This work might encompass memories from early education of being seen as ‘naughty’ or ‘stupid.’ Neurodivergent clients in particular may recall being told that they are not behaving like their peers, that they are not trying hard enough, or may have had their failings repeatedly highlighted by educators. Familiar stories are of not fitting in at school and not being ‘good enough’ as they are. Cumulative past experiences evoking feelings of threat and shame incentivise masking behaviour (e.g., suppressing stimming, forcing eye contact, copying others’ behaviours) which is increasingly recognised as a driver of poor mental health in neurodivergent people (Miller et al., 2021).

For a person to feel compassion for self and others, they need access to compassionate memories, i.e., memories of an interpersonal interaction with another who is warm, non-judgmental, sensitive to and tolerant of differences. They also need access to ‘safe spaces,’ places of welcome, belonging and enjoyment (Lucre and Clapton, 2020). These are experienced and consequently become expected. Such memories may be difficult for neurodivergent individuals to bring to mind, given the dominant societal frameworks of deficit and exclusion (Botha et al., 2020). Within psychological therapy there is an increasing focus on encouraging the individual to thrive with difference, *without* intending to treat or reduce the expressions of neurodiversity (McVey et al., 2021); compassion-focused therapies are increasingly indicated for neurodivergent clients (Robinson, 2018). This approach can serve as a useful model for education.

Important tenets of compassion in psychological therapy include warmth from the self to the self, and warmth from others (Gilbert and Bailey, 2000). In contrast, an attack and counter-attack dynamic can be at play for neurodivergent students, whereby feeling misunderstood causes them to respond with annoyance or withdrawal from the education system. ‘Winning,’ or asserting their learning needs, for the more proactive students will not necessarily resolve the difficulties according to concepts of compassion, because warmth and support are missing. Furthermore, self-compassion requires a desire to grow, to look forward with hope for success and to build on positive attributes of the self. Neurodivergent students’ self-belief and ambitions for employment post-graduation are often challenged by negative experiences of academic discrimination (Cheriyan et al., 2021). Within schools and universities, where being neurodivergent often makes you a more ‘troublesome’ student, thriving is typically curtailed, with negative implications for future hopefulness. Implementing concepts from the foundational underpinnings of compassion-focused therapy (Gilbert, 2007) in educational contexts holds promise, if institutions can establish a shared intention to include all students and recognise the mutual benefits that arise from doing so.

We can borrow practical recommendations from other sectors, which are revising their ways of working collaboratively with neurodivergent clients, to develop the quality of our interactions in higher education. Maddox et al. (2020) have drawn attention to the barriers and facilitators to therapeutic working that tend to be put in place by clinicians, care coordinators and service managers within healthcare settings. Their recommendations for clinicians working with autistic clients include: clearly explaining what clients can expect and what is expected of them; establishing mutual understanding of what is being spoken and what is being implied; providing structure for in-person sessions and independent tasks; and considering ways to limit time spent in crowded, brightly lit, or noisy spaces, such as waiting rooms. Considerations for different neurodivergent client groups include the use of planned breaks and switching between tasks to avoid prolonged attentional demand or too-challenging stretches of high-priority work (Young, 2012). These good practice guidelines from healthcare can often be transferred to the classroom, allowing the unwritten curriculum to be minimised so that students can learn.

In healthcare contexts, compassionate interactions have been shown to calm, but not sedate patients. Adopting a compassionate approach does not make appointments longer and may reduce long-term patient costs, such as fewer onward referrals and better medication adherence (Trzeciak et al., 2019). If we can adopt similar principles in education, we might expect students to be more relaxed and better engaged, to have less need for supplementary study skills or wellbeing support from universities, to reach closer-to-potential academic achievement and onwards employment. These intentions are currently speculative, awaiting more investment in research.

## 5. Conceptualising compassion in educational contexts

Educators have a unique opportunity to cultivate compassion within the learning environments that we create. (Note that we advocate for universal compassion, rather than compassion specifically for a minority.) However, the development of compassion and other ‘intellectual virtues’ receive limited recognition as legitimate learning goals in the current higher education climate (Maxwell, 2017). Instead, utilitarian values of competition, choice, independence, value for money and individual achievement dominate in many countries (Sauntson and Morrish, 2010). Waddington (2018, p. 87) argues that, in an era of marketisation, contemporary university cultures in the United Kingdom and elsewhere are often characterised by “subtle, but powerful, competition and striving for prestige and dominance … [stifling] the conditions in which compassionate pedagogy can survive and flourish.” Creating the conditions for a compassionate learning environment can therefore be highly challenging for educators charged with meeting an array of market-driven targets.

Notwithstanding this unconducive context in the sector, students, and especially those in minoritised groups, flourish in learning environments in which they feel that they belong and are valued. Similarly, educators are empowered to create compassionate learning environments where there is a compassionate leadership culture in universities (Belak and Waddington, 2021). Studies within other organisational cultures have characterised compassionate leaders as: attending to and listening with interest; seeking to understand challenges; empathising; and acting to remove obstacles and obtain resources where they are needed (West and Chowla, 2017). If these behaviours can be enacted by university leaders, a culture is established which values and supports compassionate pedagogies, to the benefit of students and staff.

Hao (2011, p. 92) characterises compassionate pedagogy as underpinned by “a commitment that allows educators to criticise institutional and classroom practices that ideologically place underserved students at disadvantaged positions, while at the same time be self-reflexive of their actions through compassion as a daily commitment.” Social, pedagogical and physical aspects of the learning environment can place neurodivergent students at a disadvantage, which often goes unnoticed. A common theme in the educational experiences of these students is anxiety, triggered by uncertainty of what is required of them in the learning situation, interactions with others inside and outside of the classroom, fear of failure, managing time, perfectionism, and additional causes of stress and fatigue not shared by their peers (e.g., sensory stress), among many other factors (Gurbuz et al., 2019; Clouder et al., 2020). It is important to note that anxiety is not a *characteristic* of neurodivergence *per se*, but rather a likely *outcome* of cumulative experiences, including marginalisation and stigma, through the lifespan. Additionally, employment struggles, financial hardship and co-occurring mental health conditions are vulnerability experiences that differ between neurodivergent and neurotypical peers (Griffiths et al., 2019). When neurodivergence contributes to students feeling anxious in the classroom, learning potential and engagement is seriously curtailed. There are negative implications for attendance, joining in when present in the classroom, sharing activities with peers, and for all measures of academic attainment (LeDoux, 1998; Jones et al., 2019).

Anxiety is in essence a response to threat. In educational contexts, threat can come from pedagogical and organisational practices that encourage students to dwell on their failings, to feel disappointed in themselves, and to doubt their futures (mirroring the threats to self-compassion as conceptualised in compassion-focused therapy; Gilbert et al., 2004). Conversely, education has the capacity to transform the lives of those who have experienced discrimination and oppression (Freire, 2006). A learning environment where difference is accepted and where each student can contribute and find a sense of belonging can reduce threat, foster self-compassion, and elicit more compassionate responses from others. These experiences build banks of compassionate memories of the self in education.

## 6. What would compassionate pedagogy for neurodivergent students look like?

Implementing the principles of compassion in higher education settings is more complex than in the context of one-to-one therapy. Educators have to balance the needs and interests of multiple stakeholders; staff workloads are perennially high, and institutional structures and processes tend to be inflexible. Nonetheless, if the challenges posed by the neurodiversity paradigm can be framed as an opportunity to rethink and improve pedagogical practice, this is likely to be to the benefit of all students. In making the following recommendations, we draw on the expertise of neurodiverse, multi-stakeholder teams of students, colleagues and authors (e.g., Spaeth and Pearson, 2021; Dwyer et al., 2022; Elsherif et al., 2022; Farrant et al., 2022).

There is a clear role for university senior leadership teams in improving experience and outcomes for neurodivergent students. Including neurodiversity as a dimension of difference in equality, diversity and inclusion (EDI) reviews and initiatives is an important starting point (Dwyer et al., 2022). Any campus-wide review, investment and enhancement activity should be driven by neurodivergent staff and students in collaboration with neurotypical allies. Since experiences of discrimination, stigma and bullying contribute to poor educational outcomes for neurodivergent students, investment in university-wide training in neurodiversity is warranted to the same extent as other campus-wide EDI training. Representation matters for student belonging and aspiration; human resource leaders can therefore ensure that hiring practices are neurodiversity-inclusive, and that neurodivergent staff in the university community are appropriately supported to fulfil their roles and progress to leadership positions. In planning for the development of campus estates, leaders can consider the sensory environment (e.g., adjustable lighting, seating selections, noise and heat levels, accessibility of quiet spaces to all members of the university community). Leaders in professional service roles can design student support services that are joined up, transparent to access and simple to navigate to reduce the self-advocacy burden on students. Providing services based on need, rather than disability diagnosis is recommended, and neurodiversity-affirming mental health support services are particularly important (McVey et al., 2021; Chapman and Botha, 2022; Petty et al., 2023). Finally, investment in transition support programmes into and out of university can impact retention of, and graduate outcomes for, neurodivergent students (Moriño and Biagiotti, 2022).

For academic staff working directly with students in the classroom, a compassion-informed approach requires that we notice distress (e.g., in relation to sensory stress or high anxiety), actively listen to neurodivergent students, are curious and empathic in our response (mindful of implicit biases and double empathy barriers), and work together with students to allow them to feel that they belong in, and can contribute to, the learning community. It is important that educators are alert to the possibility that neurodivergent students may be masking differences or difficulties, and provide alternative ways for them to communicate their needs to maximise their learning. Useful questions for educators to ask include: How much choice is there for students to demonstrate a range of skills and capabilities? How is this student learning about themselves in a way that is not deficit- or problem- focused? How is their experience in this class contributing to an accumulation of safe and positive memories of themselves in education?

In addition to noticing distress, compassionate educators notice strengths and can harness these to scaffold students’ engagement and learning. For example, a student with strong attention to detail might be assigned a role of leading on the data analysis of a research project, monitoring adherence to checklists, protocols or assessment briefs, or finalising presentation materials in a group project. Building in flexibility to assessment schedules could, for example, allow a student with high social anxiety but strong information technology skills to demonstrate their learning in an animated video as an alternative choice to a live presentation in front of an audience. Diversification of assessment types through the course of a programme of study provides all students with opportunities to excel. While narrowly prescribed ways of working are still common in many jobs, increasingly employers are recognising the value of hiring neurodiverse teams and accommodating communication differences (Krzeminska et al., 2019). Universities can do the same. Similarly, allowing for a variety of communication channels between staff and students (e.g., class discussion forums, direct messages or text-chat, in-person tutorials) accommodates communication differences and allows all students to feel part of a learning community. Students should be empowered to make personal choices about their studies that are enabled by the education infrastructure.

Designing learning to reduce anxiety would involve minimising ambiguity at all levels: ensuring materials are available in advance, utilising exemplars where appropriate, and responding empathetically to requests for clarification, as misunderstandings will almost certainly be a shared responsibility. Expectations of students would be made clear and fully explicit, for example by agreeing a class contract at the beginning of a course of study, which can be particularly helpful in reducing hidden curriculum barriers for neurodivergent students. Regularly highlighting the relevance of course content to learning outcomes (i.e., constructive alignment; Biggs, 1996) supports engagement and management of ‘information overload.’ As educators we can continuously reflect on the accessibility of our own communication, aiming for maximum clarity, concision and informativeness. Where non-literal language such as metaphor or sarcasm is used, are we providing alternative ways for students to access meaning? Are we allowing adequate time for students to process verbal instructions in the classroom, and/or supplementing with written instructions?

Important concepts of compassion include self-care, empathy and distress tolerance (Gilbert, 2007). We can learn from research that has explored ways in which neurodivergent people have described their fit-for purpose, personal ways of coping with stress and distress (Young, 2012; Bearss et al., 2016; Petty et al., 2022). To increase tolerance of distress, is a student able to modify sensory stimuli as the norm, for instance by wearing ear covers? If the physical classroom environment causes sensory stress, is there scope for students to complete tasks in a quieter environment and use online networking to check in through the class? Attendance at in-person classes is often lower in neurodivergent than other student groups for a variety of reasons; self-care might require a student to temporarily withdraw from interactions with other people. Hybrid or blended delivery could be effective in allowing students to continue to access their courses during such periods (Singh et al., 2021). More important, perhaps, is to meet students where they are in terms of attendance and increase accessibility of classroom learning in consultation with them (Spaeth and Pearson, 2021). This could include reducing attentional demands by presenting information in small chunks, building in regular breaks, finding opportunities for movement where possible, or modifying seating arrangements (Honeybourne, 2018). These recommendations reflect personal accounts of coping with distress from neurodivergent people; their implementation may reduce disadvantages associated with being in a neurominority, while maintaining the ability to receive education.

## 7. Universal design for learning

Many of the recommendations in the preceding section align with the principles of Universal Design for Learning (UDL; Rose et al., 2006; CAST, 2018). Fundamentally, UDL is an antidote to bolt-on provisions for students ‘with issues,’ who are not always well served by overworked staff and systems (Williams, 2019). Current support tends to operate on a ‘disability services’ model: assess and diagnose the student; individualise a learning support plan; add in adjustments to core teaching. This model is, perhaps unintentionally, underpinned by deficit thinking. Funding-specific support relies on diagnostic labels, necessitates onerous and lengthy processes, and places an onus on the student to advocate for accommodations. While seeking support for learning, a process which can take several months, a neurodivergent student is likely to experience a poor person-context fit at university, and may escalate from one source of support to the next while appropriate interventions are not available (Lightner et al., 2012). It is important to note that, within these structural constraints, individualised support *via* disability services can be compassionate and neurodiversity-affirmative.

As an alternative to bolt-on accommodations, we propose UDL as a compassionate pedagogy. A UDL approach to curriculum design embeds flexibility and choice in order to make learning accessible to the widest possible range of students. Within this framework, information is represented in multiple modalities (e.g., verbal explanations, visual diagrams, written text); students are enabled to express their knowledge in alternative ways (e.g., opt to prepare a written report or an oral presentation); and student engagement is scaffolded (e.g., supporting self-regulation and persistence by giving students autonomy, varying challenge level, choice, and creating a safe learning environment) (Boothe et al., 2018). The evidence base for the effectiveness of UDL for student outcomes is in its infancy. Existing research suggest that UDL is an effective methodology for improving the learning *process* for all; students undertaking UDL courses report higher satisfaction, greater engagement, and reduced barriers to learning (Capp, 2017; Soek et al., 2018). Whether effects transfer to improved attainment and reduced attrition is still to be determined.

If curricula are proactively designed for – and in consultation with – diverse learners, it is possible to move away from the reliance on students disclosing their diagnosis and having to self-advocate for individualised support for learning. Such an approach enables universities to become more neurodiversity-inclusive, while reducing demands on over-stretched student support services.

## 8. Employability and post-university

Before concluding, it is worth considering one of the many impactful outputs of accessible and compassionate pedagogic practice; that is, a carry-forward of positive academic experiences in terms of student self-esteem, hopefulness and preparedness for the workplace (Kuriyan et al., 2013; Cheriyan et al., 2021). These conditions occur when students find their place and their strengths in educational settings. Importantly, employers are starting to see the competitive advantage of a neurodiverse workforce and to consider ways to make workplaces more inclusive (Kirby and Smith, 2021). Overlapping with the application of UDL in education, the following examples of recommended good practice are from accounts of successful employment of a neurodiverse workforce. Recommendations for employers include: offering flexible working hours where possible, considering choice of seating in the workplace, allocating tasks based on employee strengths, explicitly welcoming neurodiversity in the workforce through recruitment and hiring processes, and naming a contact person for consistency and clarity of communication (Gordon and Fabiano, 2019; Maras et al., 2021; Remington et al., 2022).

Nonetheless, the current disappointing reality for the majority of neurodivergent students is to expect lower likelihood of graduating, accessing postgraduate education, finding appropriate employment that matches their skills and abilities, and enjoying stability in employment (Scott et al., 2015; Gordon and Fabiano, 2019; Moriño and Biagiotti, 2022). Success in employment builds on an accumulation of positive past experiences, in education and previous employment. In this respect, ‘employability’ holds onto the coattails of educational practices. The techniques discussed through this paper provide ways in which educators can help neurodivergent students to transition their identity to the workplace, notably at a time of anxiety and when delving into a difficult-to-imagine future (Vincent, 2019). There are ample opportunities to transfer learning of good practice from education through to employment.

## 9. Discussion and conclusion

Universities and other higher education institutions ought to be an ideal context for neurodivergent flourishing. Studying at post-secondary level allows for increased independence, autonomy and self-advocacy, for example through the opportunity to follow passions and subjects of focused interest; choice over how, when and where to do independent study; increased flexibility of routines; and opportunities to establish new peer groups around shared interests, all within a relatively safe space. On average, university settings likely exhibit greater tolerance of difference than other contexts, both preceding and following higher education. Universities can therefore play an important role in promoting lifelong wellbeing, holistic identity development and skill learning for neurodivergent students. Collectively, day-to-day experiences of interpersonal interactions that model sensitivity, tolerance and kindness, as opposed to misunderstanding and negative judgement, can be personally transformative and lead to improved employment outcomes.

However, we know that not all neurodivergent students thrive at university, and the barriers to thriving are complex. Many experience high anxiety about exposing their difference, within systems and processes that highlight deficit and put the onus on the student to obtain support. The hidden curriculum can disproportionately exclude neurodivergent students. High demands on language, literacy, numeracy, executive functioning and social interaction in university courses pose specific challenges for individuals, depending on their profile of strength and difficulty. Pedagogical practices that are not designed for cognitive diversity inhibit students from developing a sense of competence and belonging in the learning community. Some neurodivergent students may also require enhanced support to successfully negotiate the transition from home to university, extracurricular aspects of campus life, and the transition from post-secondary education to the workplace, but such support is not universally available. In combination, these barriers can prove overwhelming, with implications for attrition, wellbeing and graduate outcomes.

The neurodiversity paradigm moves the focus for change to contexts, in contrast to the historically dominant medical model assumption that interventions should target deficits in the individual. This shift is beginning to play out in therapeutic, healthcare and educational settings, but there is more work to do. The challenges of educating a neurodiverse student body (and embodying a neurodiverse staff team, through recruitment and mentoring) can be reframed as an opportunity to transform practice. Educators and educational leaders can proactively design courses and systems that are neurodiversity-affirming and neurodiversity-inclusive. At present, pockets of good practice in universities tend to occur serendipitously, rather than as a result of strategic planning. Considering neurodiversity as a dimension of EDI that overlaps with, but is not synonymous with, disability would be an important step towards making university communities more inclusive for their neurodivergent members.

In this article, we have argued that the concept of compassion, as applied in psychological therapies, offers a useful template for educators working with neurodiverse student populations. In practice, this could mean noticing students’ distress in the classroom and acting to alleviate it, harnessing individual strengths to increase students’ sense of competence, and implementing principles of UDL to increase flexibility in how students access course content and demonstrate their learning. These strategies can help neurodivergent students to build a positive schema of self-in-education, which can feed forward to post-university settings and reduce the harmful impetus to mask difference. Society benefits from cognitive diversity in myriad ways; we call on university leaders and educators to act to make higher education a more compassionate context for neurodiversity.

## Author contributions

LH conceptualised and wrote the first draft of the manuscript. SP contributed to conceptualisation and wrote sections of the manuscript. Both authors contributed to manuscript revision, read, and approved the submitted version.

## Conflict of interest

The authors declare that the research was conducted in the absence of any commercial or financial relationships that could be construed as a potential conflict of interest.

## Publisher’s note

All claims expressed in this article are solely those of the authors and do not necessarily represent those of their affiliated organizations, or those of the publisher, the editors and the reviewers. Any product that may be evaluated in this article, or claim that may be made by its manufacturer, is not guaranteed or endorsed by the publisher.
