# Corrigendum: Compassionate pedagogy for neurodiversity in higher education: a conceptual analysis

**DOI:** 10.3389/fpsyg.2023.1345256

**Published:** 2024-02-20

**Authors:** Lorna G. Hamilton, Stephanie Petty

**Affiliations:** School of Education, Language and Psychology, York St John University, York, United Kingdom

**Keywords:** neurodiversity, higher education, compassion, double empathy, Universal Design for Learning

In the published article, Spaeth and Pearson (2021) was cited fewer times than is merited. The citation has now been inserted in 2. Neurodivergent students at university, paragraph 3. The corrected paragraph is shown below.

“We suggest that current educational contexts largely illustrate a conditional view of an acceptable student, i.e., a student is acceptable only when fitting to a neurotypical standard (Spaeth and Pearson, 2021). Difference is often construed as negative, and only rarely understood as demonstrating novelty, originality or excellence in academia. For many neurodivergent students, experiences of personal confusion, navigation of multiple identities and labels through which to view themselves, and experiences of bullying and marginalisation, are all threats to holding a robust, compassionate view of the self within a university environment. Understandably, students often assume high personal responsibility for trying to make a success of their education. Many neurodivergent students describe having to act as self-advocates in order make others understand their difference, which can contribute to disenfranchisement from university communities (Fabri and Andrews, 2016; Elias and White, 2018).”

The citation has also been inserted in 6. What would compassionate pedagogy for neurodivergent students look like? paragraph 6. The corrected paragraph is shown below.

“Important concepts of compassion include self-care, empathy and distress tolerance (Gilbert, 2007). We can learn from research that has explored ways in which neurodivergent people have described their fit-for purpose, personal ways of coping with stress and distress (Young, 2012; Bearss et al., 2016; Petty et al., 2022). To increase tolerance of distress, is a student able to modify sensory stimuli as the norm, for instance by wearing ear covers? If the physical classroom environment causes sensory stress, is there scope for students to complete tasks in a quieter environment and use online networking to check in through the class? Attendance at in-person classes is often lower in neurodivergent than other student groups for a variety of reasons; self-care might require a student to temporarily withdraw from interactions with other people. Hybrid or blended delivery could be effective in allowing students to continue to access their courses during such periods (Singh et al., 2021). More important, perhaps, is to meet students where they are in terms of attendance and increase accessibility of classroom learning in consultation with them (Spaeth and Pearson, 2021). This could include reducing attentional demands by presenting information in small chunks, building in regular breaks, finding opportunities for movement where possible, or modifying seating arrangements (Honeybourne, 2018). These recommendations reflect personal accounts of coping with distress from neurodivergent people; their implementation may reduce disadvantages associated with being in a neurominority, while maintaining the ability to receive education.”

The authors apologize for this error and state that this does not change the scientific conclusions of the article in any way. The original article has been updated.
